# Getting Insights in Stakeholder Collaboration in the Transition Toward Safe and Sustainable Food Production: Net-Map Analysis of the Italian Wheat Supply Chain

**DOI:** 10.3390/foods14050786

**Published:** 2025-02-25

**Authors:** Biancamaria Ciasca, Nunzia M. Cito, Veronica M. T. Lattanzio

**Affiliations:** Institute of Sciences of Food Production, National Research Council of Italy, Via Amendola 122/O, 70126 Bari, Italy; nunzia.cito@ispa.cnr.it (N.M.C.); veronicamariateresa.lattanzio@cnr.it (V.M.T.L.)

**Keywords:** food chain sustainability, multi-actor approach, wheat supply chain

## Abstract

The transition toward sustainable food systems is a critical challenge for the European Union (EU), requiring integrated policies and collaborative action. However, the complexity of food systems and the diverse interests of stakeholders often hinder effective progress. To apply a systemic approach and deliver context-specific solutions, it is necessary to establish key commodity/supply chain groups that include diverse perspectives from multiple stakeholders. Focusing on the Italian wheat supply chain, this research addresses the gap in understanding stakeholder relationships and their influence on the definition and implementation of sustainability measures. It introduces Net-Map analysis as a fit-for-purpose, multistakeholder, participatory approach to map and analyze these relationships, identifying key actors, power dynamics, and opportunities for fostering collaboration. Through a participatory workshop and in-depth interviews, the study gathered data providing insights into stakeholder roles and leverage points. The findings contribute to improved stakeholder involvement in decision-making processes, ultimately supporting the development of a more resilient and sustainable wheat supply chain in Italy.

## 1. Introduction

Reaching sustainable food systems requires building sustainability culture, setting an enabling policy framework, and taking collective actions. A harmonized legislative framework would allow food systems’ stakeholders across EU countries to jointly respond to sustainability demands set in a number of policies, seeking synergies while respecting local and context-specific situations. In this respect, the EU policy initiative for a Legislative Framework on Sustainable Food Systems [1] is expected to play a pivotal role in accelerating the transition toward sustainable food production. The proposal for regulation should lay down general principles and objectives, together with the requirements and responsibilities of all actors in the EU food system. It should ensure coherence with all EU food-related policies (e.g., agriculture, fisheries, and aquaculture) in terms of sustainability objectives, including biodiversity and climate objectives.

The proposal was expected to be tabled in the fourth quarter of 2023. To date, it is not mentioned on the tentative agenda for forthcoming Commission meetings or in the Commission work program for 2024. The timeline for presenting this proposal is still undetermined, likely due to the complexity of the issue given its requirements for a multidimensional and integrative approach that considers all relevant actors.

Shifting to sustainable food production requires food safety attention, even more, due to new food system transformation practices targeting circularity, diversity, etc. [2]. The Farm to Fork Strategy, as a key element of the European Green Deal, recognizes that food safety and security are at the core of sustainable food systems [3]. Bené et al. [4] highlighted this aspect in their rigorous analysis of the metrics/indicators for food system sustainability. Four dimensions are widely considered when exploring sustainable food systems: environmental, economic, social, and food/nutritional. The food/nutritional dimension may be further categorized to include the food safe subdimension, along with food security, waste/losses, and nutrition.

Due to the multidimensional nature of food systems sustainability and the related indicators, coordinated action and collective efforts by multiple stakeholders along the supply chain are essential to unravel and address challenges in an integrative manner [5] One of the primary gaps to be addressed for effective implementation of sustainability measures is the alignment of stakeholders around a common vision and goals, transcending their specific sectors.

Recent studies have shown a growing focus on the implications and challenges of establishing meaningful collaborations among the diverse range of stakeholders in sustainable food supply chains. This topic has been extensively discussed by Dania et al. (2018) [6], unravelling both vertical and horizontal collaboration approaches. Vertical collaboration, which is the focus of the present study, occurs between actors at different levels of the supply chain (e.g., farmers, processors, retailers), while horizontal collaboration involves actors at the same level (e.g., farmer cooperatives) [7]. The review by Dania et al. (2018) [6] highlights how vertical collaboration is crucial in food supply chains since it improves traceability systems, quality control mechanisms, and the efficiency of resource allocation and enhances the information flow from farm to fork. A quite comprehensive bibliometric analysis of the topic “Collaboration for the sustainable food supply chain”, built on a sample of 528 articles, has been reported by Duong et al. (2023) [8]. This analysis reveals a consensus on the need for collaboration due to the complexity of the food industry and the nature of the food products. Some gaps to be covered by future research have been identified, including the need for more studies to explore ways to engage stakeholders in the sustainability transition, with a stronger focus on social sustainability, and in the emerging economies context. A promising approach to foster stakeholder engagement has been proposed by Ammirato et al. (2021) [9] who introduced the concept of network-based collaboration, combining both vertical and horizontal elements. The authors see this approach as an effective strategy to help supply chain actors in reaching sustainability goals, since it creates multi-stakeholder spaces for dialogue, facilitates knowledge exchange across the entire supply chain, enables collective problem-solving, and promotes innovation through diverse perspectives.

A specific focus on collaboration schemes for sustainability practices’ implementation in Italian supply chains can be found in León-Bravo et al. (2017) [10]. Their findings suggest that sustainability performance, in several areas including sustainable sourcing and green processing, packaging, transportation, and social sustainability can be associated with some collaborative practices implemented in the upstream and/or downstream. With respect to vertical collaboration, the study also clearly shows the heterogeneity of relationships established among actors for sustainability purposes, concluding that no single standard frame can be used to manage them.

Establishing key commodity/supply chain groups that include diverse perspectives from multiple stakeholders is necessary for delivering more innovative and context-specific solutions [11]. The wheat supply chain in Italy comprises numerous stakeholders, including farmers, processors, and retailers, but it remains fragmented. Different stakeholders have varying priorities and levels of commitment to sustainability, which complicates collaborative efforts. For example, while some companies may prioritize sustainability for brand enhancement, farmers often focus more on immediate economic concerns. Effective coordination among these parties is crucial for implementing sustainability measures; however, existing communication channels are frequently inadequate, leading to inefficiencies and misunderstandings about sustainability goals [12]. This situation can result in limited participation in the co-creation of knowledge and practices related to sustainability.

To cope with this fragmentation, a first step is represented by the characterization and assessment of multi-stakeholder roles and connections. In this context, Net-Map analysis has been proven to be a fit-for-purpose tool. The Net-Map methodology is an interview-based mapping tool that involves many categories of stakeholders (e.g., policy, food business, society, and science) within a given system in a structured participatory process. It allows for (1) visualizing formal and informal networks within the defined system, unravelling relationships, spheres of influence, and objectives; (2) discovering sources of conflicts and possibilities for cooperation; (3) facilitating knowledge exchange and mutual learning processes to ultimately foster the development of visions and strategies to achieve common goals. It was originally developed to map multiple social relationships for a diversity of purposes, such as understanding knowledge flows in governance networks or detecting actors that have influence in certain governance processes based on their position in the network [13,14]. This analysis can facilitate the understanding of some national (or regional) levels of connections between stakeholders, which sometimes may not be fully integrated, due to different roles and rigid institutional functions. The Net-Map analysis has been also implemented to support the evaluation of the City Region Food System to identify the key actors involved in the transformation of the local food system [15].

The Net-Map methodology, adapted in a digital version, has been implemented in a recent study to analyze multi-stakeholder networks in risk analysis of emerging food safety issues [16]. The study resulted in complex Net-Maps, visualizing how the science–policy–society collaboration system regarding risk analysis of the selected food safety issues (food contact materials) is set up for four target EU countries. The maps allowed us to identify the most prominent stakeholders who take up a central role in the risk analysis process as well as linkages between them. A further application of Net-Map analysis to food safety systems has been reported by Pilamala Rosales et al. [17] to gain insights on stakeholders’ roles and influence in the safety of the street food system at local markets in Ecuador.

In the present study, Net-Mapping was used to analyze stakeholder relationships within the Italian wheat supply chain. The overall mapping process was based on a participatory workshop complemented by in-depth interviews to validate and integrate the collected data. This methodology helped identify and visualize these relationships and how they influence stakeholder positioning and their role in defining and implementing sustainability indicators. The analysis of these stakeholder maps allowed us to envisage potential barriers and opportunities related to the visualized connections. Building on each participating stakeholder’s unique knowledge and perspective, this method supports more robust multi-stakeholder collaborations aimed at orienting the national wheat supply chain toward long-term sustainability.

## 2. Materials and Methods

### 2.1. Study Participants

The collaborative exercise (online workshop and in-depth interviews) involved 14 experts covering the following stakeholder categories: science (academia and research), policy (policy officers), food business (small and medium enterprises), and society (associations). The anonymized list of the Net-Map analysis (participatory workshop and in-depth interviews), their role in the wheat supply chain and relevant expertise is reported in Table 1.

### 2.2. Net-Map Workshop

The applied Net-Map methodology is based on recent adaptations developed and tested by Pilamala Rosales et al. [17] and van der Linden et al. [16]. Before conducting the actual Net-Map analysis, as recommended by Schiffer (2007) [13], some background information on stakeholders’ categories and goals, here called preliminary analysis, were collected.

**Figure 1:**
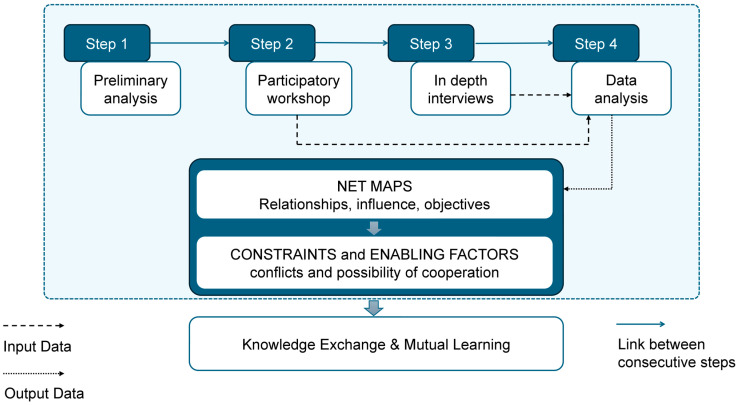
Steps of the Net-Map methodology.

The resulting methodology, tailored to the selected case study, is described in Figure 1.

Briefly, after the preliminary analysis, the participatory workshop complemented by in depth interviews allowed for (i) building the Net-Maps by identifying and categorizing the key stakeholders who play a role in the network, identifying relationships that connect them and consider domains of influence (e.g., information, data, cost flows); (ii) envisaging barriers and enabling factors in relation to the visualized connections in the Net-Maps.

To guide the participants in providing their contributions in a structured pathway, a digital board was designed using the Miro tool (https://miro.com/it/, accessed on 12 November 2024). The Net-Map protocol was used to create a whiteboard with all required frames for each step, including the task description (see Supplementary Material S1). A training session was planned the week before the workshop to ensure smooth participation and make participants confident with the use of the digital board.

Details of each step are given in the following.

#### 2.2.1. Step 1—Preliminary Analysis

A preliminary analysis that involved scanning available literature and sustainability guidelines/statements available from food business operators was carried out to define terminology, to draft an initial list of sustainability indicators, and to identify the network members and the linkage types [4,18,19,20,21].

#### 2.2.2. Step 2—Collaborative Exercise on the Digital Board

The participatory workshop comprised four phases, as described below.

A1. Stakeholders’ identification: A provisional list of the stakeholders and their role was provided to the participants (see Supplementary Material S1—board 5). Participants were subsequently asked to revise and integrate it.

A2. Characterization of the relations between the stakeholders and their character: Participants were asked to connect the identified stakeholders with arrows of three different colors based on the three different linkage types: (a) red arrows for legally required information and data sharing, (b) green arrow for voluntary information and data sharing, and (c) blue arrow for costs flow. The term “data” included metric measures of the sustainability indicator, whereas “information” meant origin and social indicators.

This step led to the construction of Net-Maps.

A3. Identification and prioritization of actual or potential constraints: In relation to the visualized connections in the Net-Maps and taking into account four categories of constraints, namely (1) data: lack of data sources, lack of detail at the supply chain/regional level, and the shortage/absence of methodologies for data processing; (2) capabilities: shortage/absence of obligations (legislative or disciplinary); (3) resources: shortage/absence of funds, human resources, time, knowledge, or experience; (4) relations: shortage/absence of contacts, non-functional/non-operational relationships, and participants were asked to indicate a maximum of two main constraints for each stakeholder category, where they thought it relevant.

A4. Identification and prioritization of actual or potential enabling factors: In relation to the visualized connections in the Net-Maps, participants were asked to envisage potential enabling factors. Once a list of enabling factors was drafted collectively, the participants were asked to individually rank each listed enabling factor by assigning the following scores: 1 = low impact enabling factor, 2 = medium impact enabling factor, and 3 = high impact enabling factor.

#### 2.2.3. Step 3—In-Depth Interviews

To validate and integrate the collected data, five further experts were involved in bilateral in-depth interviews. For this purpose, step 2 exercises were reported in the form of questions and tables to be filled in by participants (see Supplementary Material S2).

#### 2.2.4. Step 4—Data Analysis

Data from the activity A2 of step 2 (participatory workshop) and relevant questions/tables in step 3 (in depth interviews) were entered into Microsoft Excel worksheets. Four separate worksheets corresponding to four symmetrical square matrices were generated for each link type, namely (1) information required by law and data sharing, (2) voluntary information and data sharing, (3) cost flow, and (4) combined links including all the previous link types between the stakeholders. In these matrices, all stakeholders were arranged in the same order in columns and rows. A score equal to “1” indicated a link, while a score of “0” indicated no link. The score equal to 1 indicated either a mono directional or a bidirectional link. In the matrix of combined links, when two actors are connected to each other by links of a different type, the link is counted only once.

The three worksheets were imported into yEd (version 3.21.1, yWorks GmbH, https://www.yworks.com/products/yed/download, accessed on 2 January 2025) to create the corresponding Net-Maps. The organic layout algorithm was selected to represent the Net-Map graphs. Specific centrality values (indegree, outdegree, degree, and eigenvector centrality) were calculated using Gephi version 0.10. The results were four Net-Maps in the domain of the following linkages: (1) legally required information and data sharing, (2) voluntary information and data sharing, (3) cost flow, and (4) combined links where lines and arrows reflect the relationship between the stakeholders. In each map, the node size indicates a stakeholder’s influence.

## 3. Results

### 3.1. Participatory Workshop

#### 3.1.1. Setting the Scene

##### Introduction to the EU Sustainable Food System Initiative

Understanding the legislative framework is crucial to determining the involvement and impact or role of various stakeholders in the implementation of sustainability indicators. Therefore, to provide all participants with a general understanding of current laws and future possibilities related to food system sustainability, in the first session of the workshop, an overview of the legislative framework on sustainability was given, also considering the ongoing EC initiative on sustainable food systems [22]. The main insights are summarized below.

The ambition of this initiative is to provide the EU with a wide sustainability approach to reduce not only the social and environmental impacts of food systems but also to make them more resilient. The new framework should ensure coherence with all EU food-related policies (including food safety policies). The envisaged possible policy options are based on different scenarios:

1. Baseline: This involves pursuing sustainable food systems in the context of the implementation of existing legislation and consistently with the objectives of the Farm to Fork Strategy, while taking into account the possible multitude of approaches to sustainability of food systems by Member States and stakeholders.
2. Voluntary approaches: This option foresees the assessment of whether and to which extent voluntary approaches through soft law instruments can contribute in the long-term to the transition toward sustainable food systems
3. Reinforcement of existing legislation: This involves pursuing a comprehensive transition toward an Union sustainable food system, considering the objectives of the Farm to Fork Strategy, by having a number of targeted sectoral EU level interventions through existing Union Acquis (i.e., the collection of common rights and obligations that constitute the body of EU law).
4. Building a new comprehensive legislative framework on sustainability that could serve as a *lex generalis*: This would apply to all actors of the food system. It would set out the common basis composed of general objectives, definitions, principles, and requirements for ensuring sustainability considerations, beyond the already applicable safety-based requirements set in the General Food Law (Regulation (EC) No 178/2002). This common basis will serve as an integrated general approach for *lex specialis*, when addressing specific contexts.

Referring to the existing and upcoming EU regulatory framework, experts have pointed out that numerous programs are being implemented at the national level, with each member state advancing independently in its own way. Therefore, among the key elements for the evaluation and national implementation of the possible envisaged policy options, the experts indicated the following:

- Setting sustainability principles and objectives to provide a common understanding of the goals to be achieved;
- Setting general minimum standards to be met for foods produced or placed on the EU market and related food operations;
- Establishing clear responsibilities of food system actors;
- Including horizontal elements for sustainability analysis in relation to regulated products in the food chain, complementing the existing ‘risk analysis’ principle (food safety and general food law).

##### Introduction to the Case Study

The wheat supply chain was proposed as a case study due to its crucial role in Italy’s agro-food landscape, national economy, and cultural heritage. Considering both durum and soft wheat, the total wheat production is estimated at around 4 million tons, which would account for roughly 25.9% of total cereal production [23,24]. Wheat is integral to Italian cuisine, especially in pasta and bread production. Italians consume over 100 kg per capita per year of durum and soft wheat products, which includes bread, cakes, and pizzas [25]. The supply chain of durum wheat, semolina, and pasta plays a vital role in the global image of ‘Made in Italy’ and the Mediterranean diet, particularly as a unique resource for some agricultural districts in Southern Italy [26]. Some Italian pasta producers are strongly committed enhancing the wheat value chain through collaborations to create tools that support farmers in sustainable cultivation and to address consumers’ demand for higher quality and environmentally friendly products [26,27].

##### Introduction to the Methodology

A brief introduction to the Net-Map analysis methodology is given, explaining the goals and non-goals. Specifically, the goal of the participatory exercise was to identify stakeholders of the Italian supply chain that contribute to the definition/implementation of sustainability indicators, visualize and characterize their connections, and finally reflect on constraints and opportunities in relation to the visualized connections. Identifying or proposing sustainability indicators was out of the scope of the collaborative exercise (non-goal).

#### 3.1.2. Background Information

**Figure 2:**
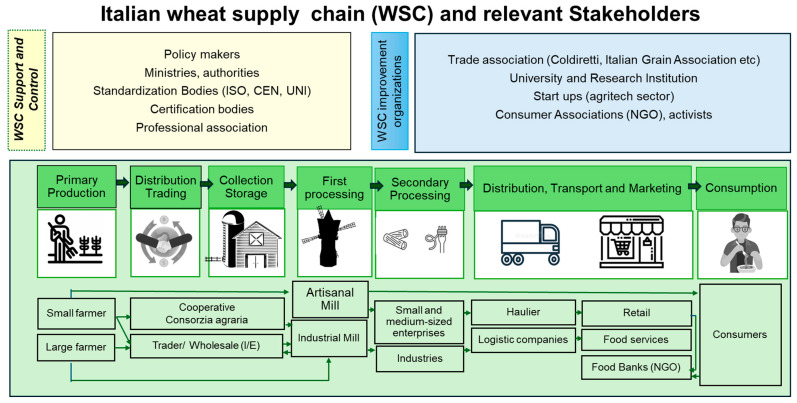
Simplified scheme of the Italian wheat supply chain and relevant stakeholders.

To bring together all participants (either in the workshop or in interviews) at the same background level and refer to a real/concrete situation when performing the collaborative exercise, a non-exhaustive list of sustainability indicators for the cereal supply chain was presented (Table 2) as well as a scheme of the Italian wheat supply chain depicting the main stakeholders (Figure 2).

#### 3.1.3. Identification of Stakeholder Roles and Linkages

After providing the above information, participants were guided through the collaborative activities.

The first activity consisted of two parallel tasks: (1) identification of the stakeholders that in Italy contribute to the definition/implementation of sustainability indicators and (2) identification of their potential contribution or actual role relevant to the definition or implementation of sustainability indicators. Starting from a draft list of stakeholders and roles provided as background material (see Section 2.2.1—Step 1), with respect to the case study “sustainable wheat supply chain”, participants identified a wide variety of stakeholders, belonging to several categories and with different roles, contributing directly or indirectly to the definition/implementation of sustainability indicators.

The final stakeholder list (Table 3), drafted during the participatory workshop and fine-tuned through bilateral interviews, showed the diversity of contributions and roles that are necessary to achieve the common overall objective or increasing the sustainability of the Italian wheat supply chain.

With a view to the incoming sustainability regulation and the possible scenarios for national implementation, the role of the “standardization bodies” and “certification bodies” was discussed. In principle, the two stakeholder categories have different although linked roles: the “standardization bodies” draft standards, while the certification bodies provide services to companies to register the sustainability standards. However, the frontier between standard setting and standard certification is increasingly blurred: many certification bodies are involved both in standard setting and certification (albeit in formally distinct guises), while some non-governmental organization traditionally engaged in defining sustainability standards now also take part in certification [28].

**Figure 3:**
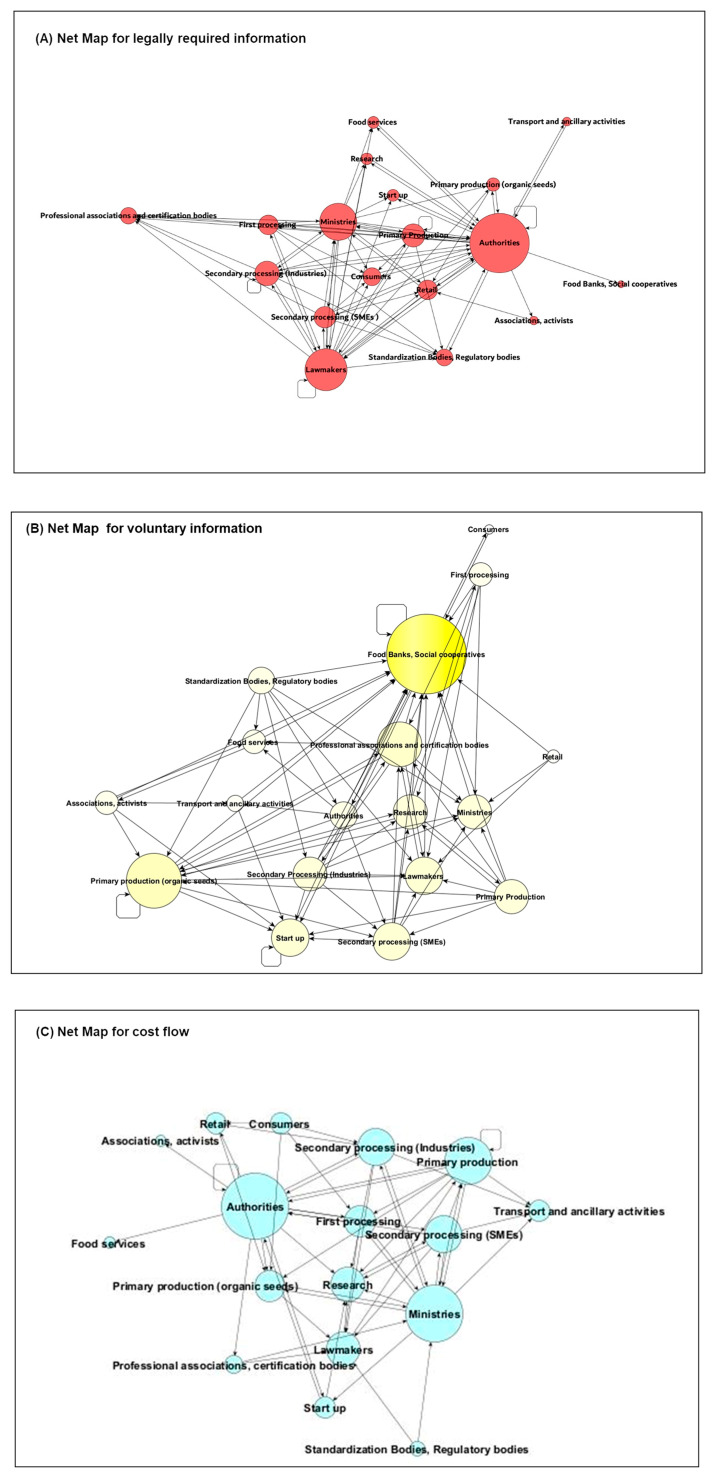
Net-Maps depicting the relationships among stakeholders of the Italian wheat supply chain contributing directly or indirectly to the definition/implementation of sustainability indicators. Arrows indicate three linkage types: (**A**) exchange of legally required information, (**B**) exchange of voluntary information, (**C**) cost flow. Node size indicates the stakeholder’s influence (total number of connections—degree centrality measure).

The second collaborative activity consisted of identifying and visualizing the connections between the stakeholders and defining the linkage type, choosing from the following: (1) legally required information and data sharing, (2) voluntary information and data sharing, and (3) cost flow. This activity resulted in the Net-Maps depicted in Figure 3A, B, and C, respectively. To build the Net-Maps, the connections visualized by colored arrows on the digital board, during the participatory workshop, were integrated with connections indicated by the experts in the in-depth interviews (see Methods Section 2.2.4—data analysis).

For the interpretation of the visual data (Net-Maps), centrality measures (Table 4) were also taken into account, since they provided quantitative information on the number of inputs that a stakeholder receives from the other stakeholders (in DG), provides to the other stakeholders (out DG), and exchanges with the other stakeholders (DG). The normalized eigenvector centrality (EV) provided information on the extent to which a stakeholder is connected to other well-connected stakeholders.

The network for legally required information and data sharing (Figure 3A) sees authorities, ministries, and law makers as the most influential actors based on the input they provide to other stakeholders (highest out DG values) and the highest number of connections they have with the most influential stakeholders in the system (highest EG values). Other influential stakeholders in this Net-Map, in terms of input provided to the system (high out DG values), are those normally interlinked by contractual/legal obligations, such as primary producers, first and secondary processors, retailers, and, indeed, authorities. On the other side, the least influential actors, showing a lower number of connections and exchanges of obligatory information within the system, are associations (DG: 0.06, EG: 0.16), transport (DG: 0.06, EG: 0.11), and food banks/social cooperative stakeholders (DG: 0.03, EG: 0.11). Consumers have a central positioning in the map but a small node size (influence), being targeted by the large amount of information received, since legally required, from policy actors and food business operators. The network for voluntary information and data sharing (Figure 3B) is quite different. As it turns out, the food bank is the most prominent stakeholder collecting information through connections with a wide diversity of actors along the supply chain (inDG value: 18, EG value: 1.00). The central role of food bank/social cooperative group is mainly due to the high number of voluntary incoming information (18 out of 22 total linkages identified), since collection of data and information is within their tasks either when operating at a national level or as an international federation. Food banks act as intermediaries by collecting surplus and redistributing grain products to vulnerable populations. For this purpose, they establish and feed a relevant information flow through cooperation with growers, millers, and food producers to source food products especially during times of surplus, reducing food waste. For the remaining stakeholders, an equal distribution between incoming and outcoming voluntary information is observed. Stakeholders from the policy side (ministries, authorities, and certification bodies) appear also quite influential in the map, given the high number of connections visualized also in the domain of exchange of voluntary information (DG values from 0.32 to 0.45). Similar DG values, i.e., overall number of connections, have been obtained for primary and secondary (processing) producers, including the small and medium enterprises (SMEs). As associations/federations, these stakeholders either support individual wheat farmers and wheat processors or act at an advocacy level to promote the overall sustainability and efficiency of the wheat sector (link with policy stakeholders). It is worth mentioning that, although not central in the system, start-ups are well connected with primary and secondary producers, indicating some commitment for innovation along the Italian wheat supply chain.

Finally, the Net-Maps for cost flow shows the prominence of the authorities (DG value of 1.00, EG value of 1.00) and the ministries (DG value of 0.83, EG value of 0.85) either investing money or receiving incomes from the implementation of sustainability measures. With one exception, this map shows mostly unidirectional flows rather than bi-directional ones. The cost flow is visualized mainly from food production actors (primary production, processing, logistics, and retail) to policy/law actors. The actors belonging to the primary production group are connected to the most influential stakeholders (EG value of 0.98), although the cost flow is direct to a limited part of the network (DG value of 0.28). Researchers show the highest EG value (EG value of 1.00) because they are well connected to ministries, law makers, and authorities, reflecting current resources allocation in research that is supposed to provide innovations to increase the sustainability of food production as well as to support technology transfer. Consumers are characterized by low DG and EG values as compared with primary/secondary producers, suggesting that the latter is the most impacted by additional production costs arising from the request to comply with sustainability standards.

**Figure 4:**
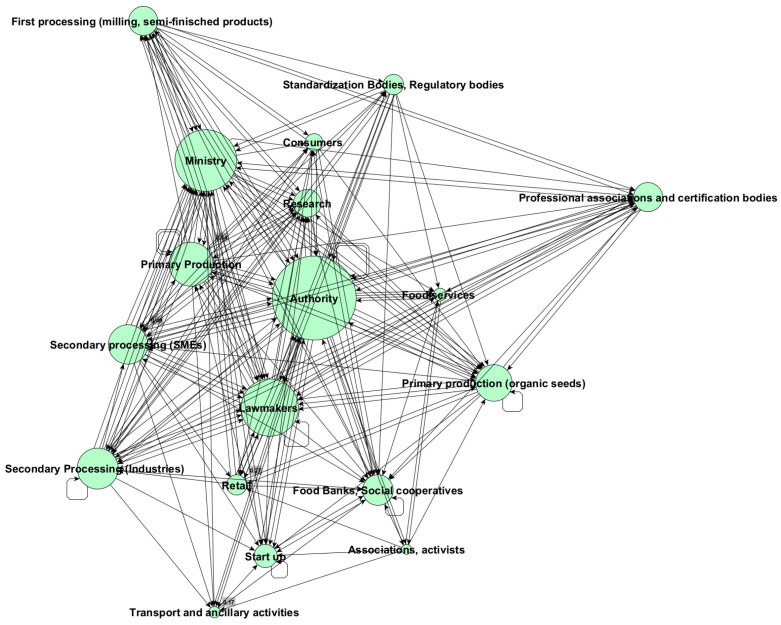
Combined Net-Map of the relationships among stakeholders of the Italian wheat supply chain contributing directly or indirectly to the definition/implementation of sustainability indicators. The map combines data obtained for the three analyzed linkage types: legally required information, voluntary information, and cost flow. Node size indicates the stakeholder’s influence (total number of connections—degree centrality measure).

To sum up, combining all three linkages in a single Net-Map (Figure 4), stakeholders from the policy side, i.e., authorities (DG value of 1.00, EV value of 1.00), lawmakers (DG value of 0.65, EV value of 0.93), and the ministries (DG value of 0.69, EV value of 0.90), reassert themselves as the most influential stakeholders in the definition and implementation of sustainability measures. They are surrounded by primary and secondary producers (EG values from 0.89 to 0.83) setting connections and influencing the system mainly through corporate entities.

In the overall picture, consumers are a much less influential stakeholder category. Consumers show a DG value of 0.40 and EV of 0.52 mainly due to their connections in the network of legally required information and data sharing, whereas they are not influential in the network of voluntary information data sharing and flow costs.

#### 3.1.4. Identification and Prioritization of (Potential) Constraints Hampering the Definition and/or Implementation of Sustainability Indicators

Starting from the Net-Maps reported in Figure 3 and Figure 4 and relying on their personal experience and knowledge in the field (Table 1), the experts were asked to further elaborate on the visualized connections and node sizes by envisaging constraints encountered by different stakeholders in the definition and implementation of sustainability indicators.

**Figure 5:**
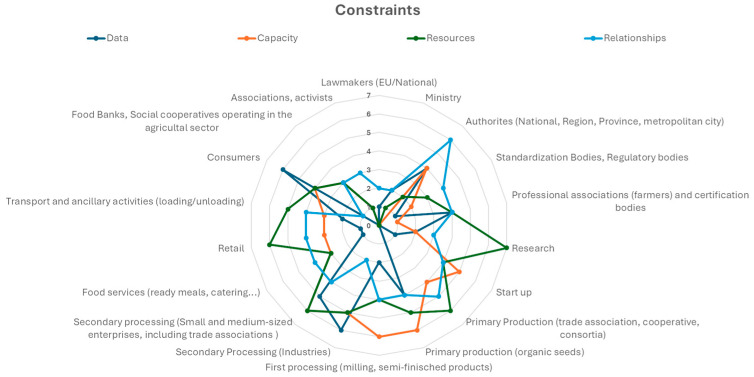
Radar graph of constraints indicated by experts hampering the definition and/or implementation of sustainability indicators by the Italian wheat supply chain stakeholders. The graph combines data obtained from the participatory workshop or the interviews. Four categories of constraints are considered: data, capabilities, resources, and relations. Each radial axis in the figure represents the number of times a given constraint was indicated (as the sum of contributions from individual participants) for each stakeholder category listed around the chart.

Figure 5 summarizes the participants’ opinions. The radar graph combines data obtained from the participatory workshop and the interviews. In both cases, participants considered four categories of constraints: (1) data: lack of data sources, lack of detail at the supply chain/regional level, and shortage/absence of methodologies for data processing; (2) capabilities: shortage/absence of obligations (legislative or disciplinary); (3) resources: shortage/absence of funds, human resources, time, knowledge, or experience; (4) relations: shortage/absence of contacts and non-functional/non-operational relationships. They were asked to identify and indicate up to two main constraints per stakeholder category where relevant. Each radial axis in the figure represents the number of times a given constraint was identified (as sum of contributions from individual participants) for each stakeholder category listed around the chart.

In general, relationships (lack of contacts and links, dysfunctional relationships) were mentioned as the areas where most improvement was needed. This constraint was reported to be particularly relevant for authorities at the national, regional, and local (municipality) level. Even though Net-Maps showed these stakeholders to be well connected and to have a high influence in the studied system, experts pointed out that relationships are often monodirectional, and there is a lack of functional/structured channels enabling the other stakeholder categories to reach them. The same consideration applies to relationships within the different geographical levels (i.e., from local to national authorities).

For industries, rather than relationships, data availability was seen as the major limitation in the definition and implementation of sustainability indicators. As shown in the Net-Maps for legally required and voluntary information exchange, the flow of data/information is mainly unidirectional, i.e., from business operators to actors responsible for drafting, implementing, or enforcing regulations, while suitable tools and channels for data exchange and integration, e.g., among business operators of the wheat supply chain, are still missing or inadequate.

Resources (finances, manpower, equipment) were identified as a major limitation mainly for researchers, followed by primary and secondary producers and retailers. All these actors are increasingly requested to allocate more effort to deliver and implement innovative solutions to make the Italian wheat supply chain more sustainable, in addition to other mandatory requirements for safety, quality, traceability, and nutrition.

Relationships were identified as limitations for authorities but not for policy makers, who Net-Maps identified as the most influential actors in the network. However, during the discussion, two further limitations attributable to policy makers emerged. These are the concrete commitment of policy makers to put into practice new sustainability measures and the difficulty of implementing them in a specific territorial context. This is particularly true when there is little propensity for change or when the implementation of indicators seems to conflict with campaign promises.

#### 3.1.5. Identification and Prioritization of (Potential) Enabling Factors Supporting the Definition and/or Implementation of Sustainability Indicators

This final step served as a basis to formulate recommendations, since it aimed at identifying and prioritizing factors enabling stakeholders to contribute to the definition and/or implementation of sustainability indicators and measures, as well as exploring opportunities and strategies for improved cooperation. Similar to the previous exercise, taking into account connections and spheres of influence visualized through the Net-Maps (Figure 3 and Figure 4) and referring to their own specific experience and knowledge in the field (Table 1), the experts were asked to identify then prioritize actual or potential enabling factors. Once a list of enabling factors was drafted collectively in the participatory workshop, the experts were asked to individually rank each listed enabling factor by assigning the following scores: 1 = low impact enabling factor, 2 = medium impact enabling factor, and 3 = high impact enabling factor. Experts contributing to bilateral interviews were asked to agree on the enabling factors list then to rank them.

**Figure 6:**
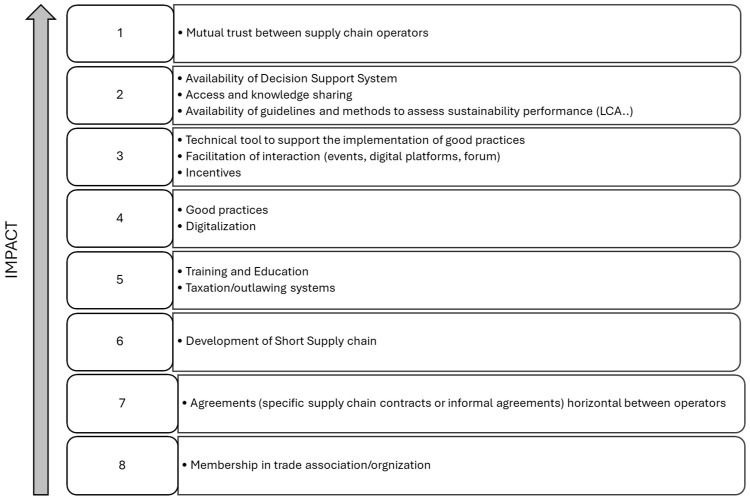
Prioritization of the identified enabling factors supporting the definition and/or implementation of sustainability indicators by the Italian wheat supply chain stakeholders.

The overall results of the ranking are reported in Figure 6. A total score ˂ 26 indicated a low impact (positions from 6 to 8 in Figure 6), a score between 26 and 28 indicated a medium impact (positions from 3 to 2), and a score ≥ 28 indicated a high impact enabling factor (position 1 in Figure 6).

The highest impact enabling factor was identified as mutual trust between supply chain operators. It was noted that this ultimate goal can be achieved only if most of the other envisaged enabling factors are properly leveraged.

Among these factors, experts prioritized the availability of practical, implementable tools such as best practices, guidelines, or decision support systems. All these tools rely on existing knowledge and data, which should be accessible to all stakeholders through dedicated cooperative spaces like digital platforms, forums, or events.

Regular participation in training and educational initiatives was highlighted as a prerequisite for efficiently accessing and utilizing available knowledge and data, as well as for generating and sharing updated resources.

Additionally, reflecting on the cost flows visualized in the Net-Map, participants emphasized that adequate incentives were essential to support and accelerate the implementation of these measures. Such incentives should be applied across various stakeholders, including supply chain operators adopting sustainability measures, innovators, and researchers developing solutions or proposing new strategies.

Regarding the different types of stakeholder connections analyzed (Figure 3 and Figure 4), formal or informal agreements between operators within a specific supply chain or segment were considered more effective than membership in trade associations or organizations. However, acting as a federation, association, or cooperative allows stakeholders to exert greater influence in the decision-making process.

## 4. Discussion

Mapping actors, roles, and relationships within a defined formal or informal network is a key step in building approaches to foster cooperation. The importance of identifying the key actors and roles for efficient engagement has been highlighted in the analysis by Ammirato et al. [9]. The reviewed case studies showed that, although food chain actors can be heterogeneous in terms of their operating environment and tasks, they can be guided to share values and culture and to re-orient themselves toward the achievement of common long-term sustainability objectives. With this in mind, the present study intentionally focused on a specific culturally and geographically relevant key commodity group, where the mapping exercise revealed a diversity of contributions and roles that are necessary to achieve the common overall objective of a sustainability-oriented supply chain.

Net-Maps of the relationships within the Italian wheat supply chain networks allowed for the visualization of the positioning and influence of each stakeholder group, showing existing asymmetries. As an example, stakeholders from the governance and policy side turned out to be the most influential group, connected with all other stakeholders mostly through unidirectional relationships. According to the analysis by Dania et al. (2018) [6], this stakeholder group can be identified as the most “powerful” in the analyzed network, having a prevalent influence on the types of information shared, recipients, and sharing mechanism in collaboration activities. The value of horizontal cooperation through associations/cooperatives emerged in leveraging voices of less influential stakeholders, such as individual wheat farmers and wheat processors. On the other hand, even though increasing consumer attention to sustainability issues was acknowledged by the experts as a driver for the implementation of sustainability measures in food production, a passive rather than an active involvement of consumers resulted in the mapped networks.

In addition to asymmetries in stakeholders’ size and positioning, the expert group also identified current limitations in cooperation. Relationships (lack of links, dysfunctional relationships) were mentioned as the areas where most improvement was needed, especially with reference to monodirectional (top-down) relationships with policy actors. As previously underlined by Stanco et al. [12] in their exploration of sustainable collective innovation along the Italian wheat supply chain, cooperation processes mostly do not take place autonomously but are supported by policies and governance mechanisms established between the players in the food supply chain. However, representative stakeholders in the Net-Map analysis advocated for bidirectional (bottom-up) communication channels to enable food supply chain actors to shape policies and governance mechanisms.

Indeed, cooperation implies the allocation of human, technological, physical, and financial resources. Therefore, in agreement with previous findings [6], overcoming the heterogeneity of resources and capabilities among stakeholders was identified as a critical challenge in establishing collaboration systems.

Trust and relationship quality emerged as the top priority among enabling factors. Fischer [29] demonstrate that long-term relationships built on trust are fundamental for successful collaboration in cereal supply chains. His analysis also indicated that trust can be significantly improved by effective communication among stakeholders. Enhanced communication and information sharing can also minimize uncertainty in production, distribution, and marketing processes [30].

In this respect, the present study identified digital platforms as the most efficient tool to foster multi-stakeholder dialogue and facilitate data and knowledge sharing, thus fostering transparency and trust. In line with the analysis by Duong et al. [8], the implementation of digital technologies to share information in a timely and accurate manner has been recognized as a key enabling factor to support collaboration toward sustainability objectives is a key enabling factor. Currently, the data shared in the food supply chain are often incomplete or inaccurate [8]. Our mapping confirms this, showing that particularly, farmers and SMEs are less involved in data sharing due to limited financial resources and necessary skills. This issue is being addressed in Italy by the ongoing research devoted to delivering an open access and integrated information system of statistical indicators regarding new technologies and methods for traceability, quality, and safety of agrifoods, including the wheat supply chain [31].

## 5. Conclusions

In this study, the Net-Map methodology was applied to analyze stakeholder relationships within the Italian wheat supply chain. The mapping process revealed a diversity of stakeholder roles and contributions. The obtained Net-Maps enabled us to visualize the interrelationships (formal and informal networks) among various stakeholders, including policymakers, food business operators, and consumers, highlighting their distinct roles and influences in driving the transition toward sustainable food systems. Starting from the analysis of the state-of-the-art of the EU sustainability framework, the participatory workshop and subsequent interviews provided valuable insights into existing constraints and enabling factors within the stakeholder network.

Based on these insights, several recommendations for actions to improve stakeholder relationships and their involvement in the decision-making process can be derived.

Considering the current state and the existing connections visualized in the Net- Maps, goals to be pursued in the short- to medium-term (0–3 years) and relevant actions include the following:

- Establishing a common language and understanding of sustainability: Participatory workshops and training sessions would address the current misalignment among stakeholders and varying priorities regarding sustainability.
- Providing spaces for building connections: This involves creating dedicated digital platforms or fora for stakeholders to share information, best practices, and discuss challenges related to sustainability that would foster and feed dialogue and collaboration.
- Facilitating knowledge exchange for innovation: This involves establishing or reinforcing networks with research institutions, agricultural extension services, and industry experts to provide technical support and promote the co-creation of knowledge and sustainable practices.
- Fostering the creation of data sources and methodologies: This includes establishing data collection, sharing, and reporting protocols for key sustainability indicators across the wheat supply chain.
- Launching small-scale pilot projects: These projects should address specific sustainability challenges (e.g., reducing water usage, improving soil health, minimizing food waste) while foreseeing multi-actor participation by farmers, processors, and retailers. This call for action, specifically addressed to funding organizations, would provide tangible examples of successful collaboration and demonstrate the benefits of sustainable practices.
- Developing and implementing incentive programs: This initiative, to be undertaken by governmental actors, should encourage farmers and processors to adopt sustainable practices, addressing their economic concerns. The development of certification schemes or labels for sustainably produced wheat products would complement this, acknowledging the producers’ commitment and addressing consumer demand for transparent information.

A long-term impact (3–5 years and beyond) is expected to be achieved by implementing the following actions:

- Advocating for supportive policies and regulations: This involves acting through federations and associations and working with policymakers to develop and implement policies and regulations that promote long-term investment in sustainability across the entire wheat supply chain. Promoting long-term multi-stakeholder partnerships would make these actions more impactful at the national and EU levels.
- Monitoring and evaluating progress: This involves developing systems for monitoring and evaluating the impact of sustainability initiatives on environmental, economic, and social outcomes based on harmonized indicators and metrics. Progress toward sustainability goals should be communicated to stakeholders and the public.

These proposed recommendations are intended to address the fragmentation and varying priorities within the Italian wheat supply chain, fostering collaboration among stakeholders and ultimately contributing to the broader goal of sustainable food systems in the EU.

*Study limitations.* The data obtained reflect the participants’ perception; this might represent a limitation of this study. Although the deep knowledge of participants and the modified Net-Map approach (participatory workshop complemented by in-depth interviews) ensure that the connections depicted in the Net-Maps reflect reality, the small size of the multi-actor group might limit the generalizability of the results. When elaborating on constraints and enabling factors, some participants did not feel fully confident in voting for other categories of stakeholders than their own, which explains the absence of data for the less represented categories (e.g., law makers). The identified barriers and opportunities are consistent with previously reported analyses focused on the Italian wheat supply chain [11,12]; however, it is worth remembering that this analysis was conducted for a specific case study within a specific national context.

## Figures and Tables

**Table 1:** Anonymized list of the Net-Map participants (participatory workshop and in-depth interviews), their role in the wheat supply chain, and relevant expertise.

Stakeholder Category	Role/Expertise
NGO—European Community of Consumer Cooperatives (COOP Europe)	Sustainability Policy Manager
NGO—European Community of Consumer Cooperatives (COOP Europe)	Communications Policy Advisor
NGO—National Food Bank (Italy)	Secretary General
Academia—University of Siena	Scientific expert—development and implementation of indicators for the sustainable development goals
Academia—University of Torino	Scientific expert—City region food system
Academia—University of Torino	Scientific expert—City region food system
Research—National Research Council of Italy	Scientific expert—Food safety and communication
Academia—University of Bari	Scientific expert—economic and environmental sustainability of agricultural practices
Academia—University of Bologna	Scientific expert—Political geography, political science, and food security
Secondary Production—Valle Fiorita, SME (bakery products)	R&D Responsiblities—quality, authenticity and sustainability of bakery products
Primary production trade association—CIA (Italian Confederation of Agriculture)	Director—support to wheat farmers for sustainability and efficiency of the wheat sector; communication to consumers
Primary production trade association—Liberi agricoltori	Farmer and Manager of Liberi Agricoltori—policy representation, financial support, and technical assistance to small and medium wheat farmers
Secondary production trade association—COLDIRETTI	President of Coldiretti Bari—promotion of sustainable farming practices, policy advocacy
Local Authority—Metropolitan city of Bari (Italy)	Manager of the Service for Protection and Valorization of the Environment, Promotion, and Coordination of Economic Development

**Table 2:** Example list of sustainability indicators for the cereal supply chain. The list was drafted from data collected in Step 1—preliminary analysis [4,19,20,21,22].

Dimension	Subdimension	Indicator Example
Enviromental	Air	Quality: gas emissions in total agriculture)
	Water	Quality: Water pH
		Use: Agricultural water withdrawal as percentage of total renewable water (%)
	Soil and land	Quality: Soil carbon content (as percentage in weight)
		Use: Agricultural land as % of arable land
	Biodiversity	Benefits of biodiversity index (0 = no diversity potential to 100 = maximum)
		Crop diversity (Shannon index)
		Absence of genetically modified organisms
	Use of energy	Agriculture and forestry energy use as % of total
		Primary energy resources—renewable (MJ)
		Primary energy resources—non renewable (MJ)
	Integrated pest management	Use of integrated pest management
Food and Nutrition	Food security	Availability: Per capita food available for human consumption (kcal/capita/day)
		Access: Per capita food available for human consumption (kcal/capita/day)
		Utilization: Access to improved water resource (% of total population)
		Stability: Price volatility index; per capita food supply variability (kcal/capita/day)
	Food safety	Traciability
		Burden of foodborne illness (number of cases)
		Deoxinivalenol content
	Food waste and use	Food loss as % of total food produced
		Hazardous waste disposed (g on 1 kg of product)
		Non-hazardous waste disposed (g on 1 kg of product)
		Radioactive waste disposed (g on 1 kg of product)
	Nutrition	Diet diversification
		Undernutrition: Stunting, children aged < 5 years stunted (%)
		Obesity: Prevalence of obesity (% of the population, over 18 y of age)
Economic		Financial performance: Agriculture value-added per worker (constant 2010 USD)
		Employment rate: Agriculture under-employment (%)
		Economic distribution: Gini index for land distribution and tendency
		Use of technological innovation
Social		Gender equality: labour force participation rate, female (% of female population ages 15+)
		Inclusion: predominant fair-trade organizations and producers
		Recognition of fair prices to farmers
		Labor condition: Management performance, parental leave, average hours of training per years per employment

**Table 3:** Identified stakeholders of the wheat Italian supply chain and their potential contribution or actual role relevant to the definition or implementation of sustainability indicators.

Stakeholder	Role/Contribution
Policy makers/Law makers	(EU) Establishing principles, definitions, responsibilities, standards, and metrics that also allow ensuring the traceability of raw materials (National) Transpose European directives and implement regulations and programming
Ministries (e.g., Ministry of Agricultural, Food and Forestry Policies, Ministry of Enterprises and Made in Italy)	Resource allocation, define strategic plans
Authorities (national, regional, and local)	May be involved in the definition of guidelines for the implementation of indicators and in monitoring sustainability performance Setting taxation, incentives, local policy-making
Standardization Bodies (ISO, NI)	Definition and development of voluntary standards, may contribute to the definition of sustainability standards
Certification bodies	Evaluate and certify compliance with sustainability standards, boost sharing of good practices and methodologies on sustainable production
Professional associations	Represent and safeguard the interests of the specific categories (farmers, enterprise) in the context of economic policy, contribute to the identification and sharing of good practices on inclusive and responsible business models in agriculture
Research	Development of innovations for sustainable production (variety selections, waste recovery strategies, packaging, logistic, food distribution) Contribution to the definition of decision support systems Development of tools, reports, surveys, etc. to support political decisions (collect measures for the definition of sustainability indicators) and market analysis
Start-ups	Development of innovations for sustainable production
Primary production (including trade associations, cooperatives, agricultural consortia)	Implementation of good practices/specific decision support systems
Primary production (organic seeds)	
First processing (semi-finished products—mills, etc.)	Assessment and selection of primary production based on sustainability performance
	Implementation of good practices/specific decision support systems
Secondary processing (industries)	Development of innovations, supply chain specifications, investment of resources Define and implement decision support systems Communication campaigns Assessment of consumer or supply chain perception (focus groups, surveys, data collection) Assessment and choice of primary production on the basis of sustainability performance Ensure transparency on conditions of use of raw materials
Secondary processing (small and medium-sized enterprises, including trade associations)	As above
Food services (ready-to-eat-meals, catering)	Assessment and choice of suppliers based on sustainability performance (short supply chains, certifications)
Retail	
Transport and ancillary activities (loading/unloading)	Optimization of logistics by intelligent transportation systems, implementation of sustainable transportation practices
Consumers	Receiving information, conscious consumption, paying for sustainable supply chains
Food banks, social cooperatives operating in the agricultural sector	Implementation of good practices/specific decision support systems Commitment for the affordability of quality and sustainable foodstuffs Donation management, good (hygienic) practices for safe handling of donations
Associations, activists	Stimulate thinking about sustainability at various levels (environmentalists, food sovereignty movements, commitment to quality food affordability, etc.)

**Table 4:** Centrality measure of Net-Maps (indegree centrality (in DG): number of inputs stakeholder receive from the other stakeholders, out DG: number of inputs stakeholder provide to the other stakeholders, normalized degree centrality (DG): number of inputs stakeholder exchange with the other stakeholders, normalized eigenvector centrality (EV): the extent to which a stakeholder is connected to other well-connected stakeholders).

Stakeholders	Legally Required Information				Voluntary Information				Flow Cost				Combined			
	In DG	Out DG	DG	EV	In DG	Out DG	DG	EV	In DG	Out DG	DG	EV	In DG	Out DG	DG	EV
Lawmakers	8	15	0.68	0.58	8	2	0.45	0.24	6	2	0.44	0.55	11	16	0.77	0.62
Ministries	7	13	0.59	0.51	8	1	0.41	0.24	7	8	0.83	0.74	10	15	0.71	0.56
Authorities	16	18	1.00	1.00	2	5	0.32	0.25	6	12	1.00	0.82	17	18	1.00	0.98
Standardization bodies	6	1	0.21	0.44	0	7	0.32	0.00	0	2	0.11	0.00	6	7	0.37	0.36
Professional associations certification bodies	6	1	0.21	0.46	6	6	0.55	0.24	1	2	0.17	0.19	8	7	0.43	0.48
Research	3	1	0.12	0.31	5	4	0.41	0.17	7	1	0.44	1.00	9	6	0.43	0.55
Start-up	3	1	0.12	0.31	8	2	0.45	0.57	2	2	0.22	0.36	11	4	0.43	0.67
Primary production	4	7	0.32	0.36	1	8	0.41	0.05	5	7	0.67	0.82	6	15	0.60	0.38
Primary production (organic seeds)	3	2	0.15	0.31	7	8	0.68	0.28	5	2	0.39	0.60	11	10	0.60	0.60
First processing	3	6	0.26	0.31	1	5	0.27	0.05	4	3	0.39	0.55	6	8	0.40	0.39
Secondary processing (Industries)	5	7	0.35	0.43	2	7	0.41	0.07	4	5	0.50	0.41	9	12	0.60	0.52
Secondary processing (SMEs)	3	7	0.29	0.31	4	6	0.45	0.17	3	6	0.50	0.59	7	12	0.54	0.45
Food services	3	1	0.12	0.31	4	2	0.27	0.14	1	0	0.06	0.19	6	3	0.26	0.34
Retail	6	3	0.26	0.42	0	3	0.14	0.00	2	2	0.22	0.19	7	6	0.37	0.42
Transport and ancillary activities	1	1	0.06	0.15	2	2	0.18	0.07	4	0	0.22	0.60	6	3	0.26	0.33
Consumers	7	1	0.24	0.52	1	1	0.09	0.25	0	4	0.22	0.00	8	6	0.40	0.52
Food banks, social cooperatives	1	0	0.03	0.15	18	4	1.00	1.00	0	0	0.00	0.00	18	4	0.63	1.00
Associations, activists	1	1	0.06	0.15	1	5	0.27	0.04	1	0	0.06	0.19	2	6	0.23	0.14

## Data Availability

The original contributions presented in the study are included in the article, further inquiries can be directed to the corresponding author.

## Supplementary Materials

The following supporting information can be downloaded at: https://www.mdpi.com/article/10.3390/foods14050786/s1, Supplementary Material S1: Boards of the participatory workshop. Supplementary Material S2: In-depth interview questions.

## Abbreviations

The following abbreviations are used in this manuscript:

SMEs	Small and medium enterprises
ISO	International Organization for Standardization
CEN	European Committee for Standardization
