# Supplementary material for: Getting Insights in Stakeholder Collaboration in the Transition Toward Safe and Sustainable Food Production: Net-Map Analysis of the Italian Wheat Supply Chain
Source: Foods. 2025 Feb 25;14(5):786. doi: 10.3390/foods14050786 (PMC11898932; doi:10.3390/foods14050786)
Supplement: Supplementary file 1 [file foods-14-00786-s001.zip › Supplementary Material S1.pdf]

Programme

- Introduction to the new regulatory framework on sustainaibility

- **Net Map Analysis**
  Step 1: introduction to case-study - cereal food chain
  Step 2: Identify the stakeholders that in Italy contribute to the definition/implementation of sustainability indicators
  Step 3: Identify the potential contribution to the definition/implementation of sustainability indicators for each stakeholder
  Step 4: Characterizing the relationships between the stakeholders
  Step 5: Identify and prioritize possible barriers to the application of sustainability indicators by stakeholders
  Step 6: Identifying and prioritising possible enabling factors for the application of sustainability indicators by stakeholders
- Conclusion

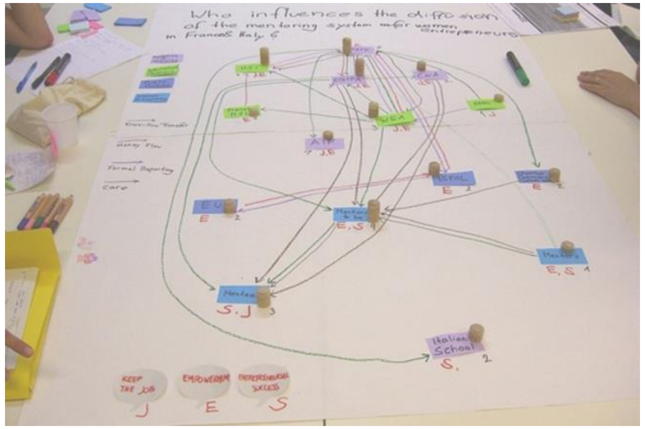

Aim of partecipative workshop

Aim

Definition and implementation of sustainability measures and relevant indicators to quantify and qualify the agrifood system.

Goal

The Net Map analysis methodology was applied to design a participatory workshop aiming at initiating a structured multi-actor dialogue to map, inform and involve stakeholders in the processes of definition and implementation of sustainability measures and relevant indicators.

No Goals

Definition of sustainability indicators.

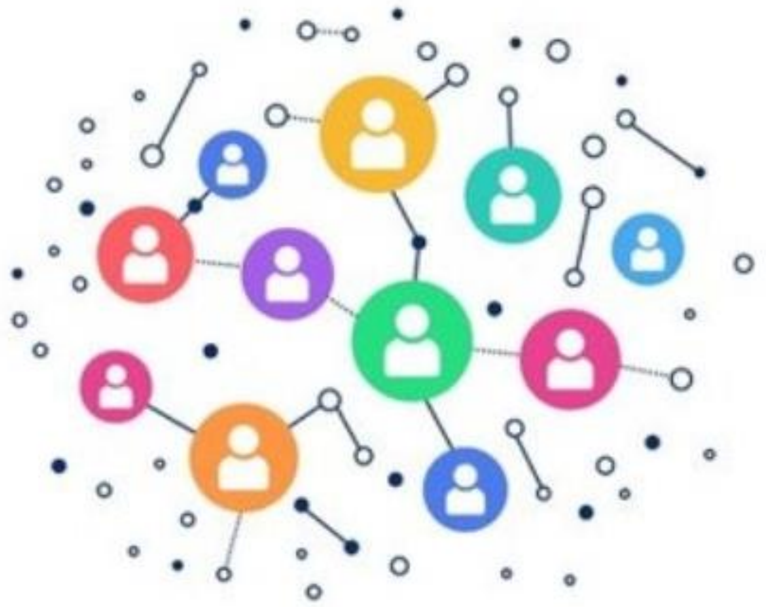

Step 1: introduction to case-study - cereal food chain

Enviromental sustainability: capacity to to conserve quality and reproducibility of natural resources - performance considers resource efficiency, recycling and pollution

Social sustainability: capacity to garantee condition for human wellbeing (human rights, labour practices, consumer protection, community involvement and development)

Economic sustainability: capacity to generate income and employment

Fourth dimension: Food Nutrition and Security

Step 1: introduction to case-study - cereal food chain

Dimension	Sub-dimension	Category	Indicators[a]
Environment	Air	Quality	Greenhouse gas emissions in total agriculture (gigagrams)
	Water	Quality	Water pH
		Use	Agricultural water withdrawal as percentage of total renewable water (%)
	Soil and land	Quality	Soil carbon content (as percentage in weight)
		Use	Agricultural land as % of arable land
	Biodiversity	Wildlife (plants, animals)	Benefits of biodiversity index (0 = no biodiversity potential to 100 = maximum)
			Crop diversity (Calories diversity measured by Shannon index)
	Energy	Use	Agriculture and forestry energy use as % of total
Economic		Financial performance	Agriculture value-added per worker (constant 2010 US$)
		Employment rate	Agriculture under-employment (%)
		Economic distribution	Gini index for land distribution & tendency
Social		Gender equity	Labor force participation rate, female (% of female population ages 15+)
		Inclusion	Predominant fair trade organizations and producers
			Employment in agriculture (% of total employment)
Food and Nutrition	Food Security	Availability	Per capita food available for human consumption (kcal/capita/day)
		Access	Food consumption as share of total income (% of total household expenditure)
			Estimated travel time to the nearest city of 50,000 or more people (Hours travel from a city)
		Utilization	Access to improved water resource (% of total population)
			Access to electricity (%)
		Stability	Price volatility index
			Per capita food supply variability (kcal/capita/day)
	Food Safety		Burden of foodborne illness (number of cases)
	Food waste and Use		Food loss as % of total food produced
	Nutrition	Diet	Diet diversification
		Undernutrition	Stunting, children aged < 5 years stunted (%)
		Overweight & obesity	Prevalence of obesity (% of the population, over 18 y of age
		Hidden hunger	Serum retinol deficiency

Examples of indicators

←

Béné, C., Prager, S. D., Achicanoy, H. A., Toro, P. A., Lamotte, L., Bonilla, C., & Mapes, B. R. (2019). Global map and indicators of food system sustainability. *Scientific data*, *6*(1), 279. https://doi.org/10.1038/s41597-019-0301-5

Step 2: Identify of stakeholders that in Italy contribute to the definition/implementation of sustainability indicators

Stakeholder	Tasks
Lawmakers	Establishing principles, definitions, responsibilities, standards and metrics
Ministry (Ministry of Agricultural, Food and Forestry Policies)	Resource allocation, strategy definition
Regional/National Authorites (Region, Province)	they may be involved in the definition of guidelines for the implementation of indicators
	they may be involved in monitoring sustainability performance
Standardization Bodies,	Implementation of voluntary standards on sustainable production.
Professional associations and certification bodies	
Research	Development of innovations for sustainable production (variety selections, waste recovery strategies....)
	Contribution to the definition of DSS
	Evaluation of consumer or supply chain perception (focus groups, surveys, data collection...)
	Market analysis
	...
Start up	Developing innovations for sustainable production
**FOOD CHAIN SKATEHOLDER**	
Primary Production (Including trade associations, cooperatives, agricultural consortia)	Implementation of good practices / DSS (specific decision support systems)
First processing (semi-finished products - mills....)	Assessment and selection of primary production based on sustainability performance
	Implementation of good practices / DSS (specific decision support systems)
Industries (Secondary Processing)	Development of innovations, supply chain specifications, investment of resources
	Definition of DSS/Implementation of DSS
	Communication campaigns
	Assessment of consumer or supply chain perception (focus groups, surveys, data collection...)
	Assessment and choice of primary production on the basis of sustainability performance
Small and medium-sized enterprises, including trade associations (secondary processing)	As above
Food services (ready meals, catering, etc.)	Assessment and choice of suppliers on the basis of sustainability performance (short supply chains, certifications....)
Retail	Assessment and choice of suppliers on the basis of sustainability performance (short supply chains, certifications....)
Transport and ancillary activities (loading/unloading)	
Consumers	Receiving information, conscious consumption, paying for sustainable supply chains
Food Banks	Implementation of good practices / DSS (specific decision support systems)
	Donation management, good (hygienic) practices for safe handling of donations
Others	

Step 3: Characterization of the relations between the stakeholders and their character

Connect the identified stakeholders with arrows of three different colors based on the three different linkage types:

legally required information and data sharing (f.i. through labeling, specifications, and requirements by national, regional regulations)

voluntary information and data sharing,

cost flow (allocation of resources, investments, willingness to pay higher costs/use of resources

Data: metric, measure of the sustainability indicator,
Information: origin, social indicators.

Options:

- Unilateral: from actor x to actor y
- Bilateral: from actor x to actor y and vice versa
- No row: no link

Example

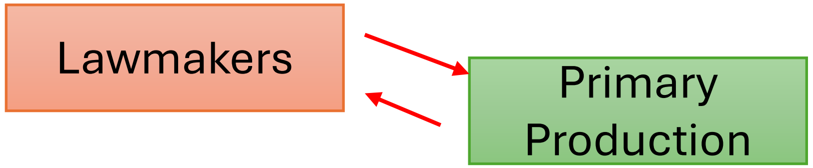

Step 3: Characterization of the relations between the stakeholders and their character:

Time: 15 minutes

→ (red) legally required information and data sharing (f.i. through labeling, specifications, and requirements by national, regional regulations)

→ (green) voluntary information and data sharing,

Ministries (Ministry of Agricultural, Food and Forestry Policies)

Standardization Bodies,

Professional associations and certification bodies

Lawmakers

Start up

Regional/National Authorities (Region, Province)

First processing (semi-finished products - mills....)

Consumers

Research

Primary Production (Including trade associations, cooperatives, agricultural consortia)

Transport and ancillary activities (loading/unloading)

Industries (Secondary Processing)

Small and medium-sized enterprises, including trade associations (secondary processing)

Food services (ready meals, catering...)

Retail

Food Banks

Step 3: Characterization of the relations between the stakeholders and their character:

Time: 15 minutes

⟶ cost flow (allocation of resources, investments, willingness to pay higher costs/use of resources

Ministries (Ministry of Agricultural, Food and Forestry Policies)

Standardization Bodies,

Professional associations and certification bodies

Lawmakers

Start up

Regional/National Authorities (Region, Province)

First processing (semi-finished products - mills....)

Consumers

Research

Primary Production (Including trade associations, cooperatives, agricultural consortia)

Transport and ancillary activities (loading/unloading)

Industries (Secondary Processing)

Small and medium-sized enterprises, including trade associations (secondary processing)

Food services (ready meals, catering...)

Retail

Food Banks

Step 4: Identification and prioritization of actual or potential constraints to the application of sustainability indicators by stakeholders

(1) data (lack of data sources, detail at the supply chain/territory level, lack/absence of methodologies for data processing)
(2) capabilities (lack of mandate to make decisions - lack of knowledge, expertise),
(3) resources (insufficient finances, manpower, equipment, time etc.)
(4) Relations (absence of contacts or links, relations that do not function properly).

Time: 30 minutes

Lawmakers

Ministries

Authorities

Standardization Bodies

SMEs (secondary processing)

Start up

Research

Food services (ready meals, catering…)

Primary Production

First processing

Industries (Secondary Processing)

Transport and ancillary activities

Professional associations and certification bodies

Retail

Consumers

Food Banks

Relations

Data

Capabilities

Resources

- **Step 5: Identifying and prioritizing enabling factors for the application of sustainability indicators by stakeholders**

Assign score to prioritize the enabling factors according to their impact on the actors contributions

1: low impact

2: medium impact

3:high impact

Technical tools to supoort the implementation of good practices

Good practices (handbooks, guidelines..)

Availability of Decision Support System

Education and training

Access and knowledge sharing

Membership in trade association/organization

Mutual trust between supply chain operators

Facilitation of interaction (events, digital platform, forum..)

Agreements (specific supply chain contracts or informal agreements) horizontal between operators

Incentives

Taxation/ outlawing system

Digitalization

Availability of guidelines and methods to assess sustainability performance (LCA..)

Development of Short supply chain
