# Supplementary material for: Getting Insights in Stakeholder Collaboration in the Transition Toward Safe and Sustainable Food Production: Net-Map Analysis of the Italian Wheat Supply Chain
Source: Foods. 2025 Feb 25;14(5):786. doi: 10.3390/foods14050786 (PMC11898932; doi:10.3390/foods14050786)
Supplement: Supplementary file 1 [file foods-14-00786-s001.zip › Supplementary Material S2_questions.pdf]

1) **Identify the stakeholders that in Italy contribute to the definition/implementation of sustainability indicators**

Stakeholder	Tasks
Lawmakers	Establishing principles, definitions, responsibilities, standards and metrics
Ministry (Ministry of Agricultural, Food and Forestry Policies)	Resource allocation, strategy definition
Regional/National Authorites (Region, Province)	they may be involved in the definition of guidelines for the implementation of indicators
	they may be involved in monitoring sustainability performance
Standardization Bodies,	Implementation of voluntary standards on sustainable production.
Professional associations and certification bodies	
Research	Development of innovations for sustainable production (variety selections, waste recovery strategies....)
	Contribution to the definition of DSS
	Evaluation of consumer or supply chain perception (focus groups, surveys, data collection...)
	Market analysis
	…
Start up	Developing innovations for sustainable production
**FOOD CHAIN SKATEHOLDER**	
Primary Production (Including trade associations, cooperatives, agricultural consortia)	Implementation of good practices / DSS (specific decision support systems)

First processing (semi-finished products - mills....)	Assessment and selection of primary production based on sustainability performance
	Implementation of good practices / DSS (specific decision support systems)
Industries (Secondary Processing)	Development of innovations, supply chain specifications, investment of resources
	Definition of DSS/Implementation of DSS
	Communication campaigns
	Assessment of consumer or supply chain perception (focus groups, surveys, data collection...)
	Assessment and choice of primary production on the basis of sustainability performance
Small and medium-sized enterprises, including trade associations (secondary processing)	As above
Food services (ready meals, catering...)	Assessment and choice of suppliers on the basis of sustainability performance (short supply chains, certifications....)
Retail	Assessment and choice of suppliers on the basis of sustainability performance (short supply chains, certifications....)
Transport and ancillary activities (loading/unloading)	
Consumers	Receiving information, conscious consumption, paying for sustainable supply chains
Food Banks	Implementation of good practices / DSS (specific decision support systems)
	Donation management, good (hygienic) practices for safe handling of donations
Others	

2) **Identifies the actors who share data by REGULATION (labelling, information, etc.) by putting an R in the corresponding cell.**
3) **Identifies the actors who VOLUNTARILY share information by putting the letter “V” in the corresponding cell.**
4) **Identifies the actors between which there is a cost flow by putting the letter “X” in the corresponding cell**

On the column there are the actors from which the data starts, on the rows the actors who receive the data

	Lawmakers	Ministry	Regional/National Authorites	Standardization Bodies,	Professional associations and certification bodies	Research	Start up	Primary Production (Including trade associations, cooperatives, agricultural consortia)	First processing (semi-finished products - mills….)		Industries (Secondary Processing)	Small and medium-sized enterprises, including trade associations (secondary processing)	Food services (ready meals, catering...)	Retail	Transport and ancillary activities (loading/unloading)	Consumers	Food Banks
Lawmakers																	
Ministry																	
Regional/National Authorites																	
Standardization Bodies,																	
Professional associations and certification bodies																	
Research																	
Start up																	
Primary Production (Including trade associations, cooperatives, agricultural consortia)																	
First processing (semi-finished products - mills….)																	
Industries (Secondary Processing)																	
Small and medium-sized enterprises, including trade associations (secondary processing)																	
Food services (ready meals, catering...)																	
Retail																	
Transport and ancillary activities (loading/unloading)																	
Consumers																	
Food Banks																	

5) **For each actor, identify the actual or potential constraints to the application of sustainability indicators by stakeholders Select from: data (lack of data sources, detail at the supply chain/territory level, lack/absence of methodologies for data processing) , (2) capabilities (lack of mandate to make decisions - lack of knowledge, expertise), (3) resources (insufficient finances, manpower, equipment, time etc.) (4) Relations (absence of contacts or links, relations that do not function properly).**

	**Data**	**Capabilities**	**Resources**	**Relations**
Lawmakers				
Ministry				
Regional/National Authorites				
Standardization Bodies,				
Professional associations and certification bodies				
Research				
Start up				
Primary Production (Including trade associations, cooperatives, agricultural consortia)				
First processing (semi-finished products - mills....)				
Industries (Secondary Processing)				
Small and medium-sized enterprises, including trade associations (secondary processing)				
Food services (ready meals, catering...)				
Retail				
Transport and ancillary activities (loading/unloading)				
Consumers				
Food Banks				

6) **Identify the enabling factors for the application of sustainability indicators by stakeholders. Vote from 1 to 3 (1: low impact, 2 medium impact, 3 high impact)**

- Technical tools to supoort the implementation of good practices
- Good practices (handbooks, guidelines..)
- Availability of Decision Support System
- Agreements (specific supply chain contracts or informal agreements) horizontal between operators
- Incentives
- Taxation/ outlawing system
- Education and training
- Access and knowledge sharing
- Membership in trade association/organization
- Mutual trust between supply chain operators
- Facilitation of interaction (events, digital platform, forum..)
- Digitalization
- Availability of guidelines and methods to assess sustainability performance (LCA..)
- Development of Short supply chain
